# Soluble CD163 as a Potential Biomarker in Systemic Sclerosis

**DOI:** 10.1155/2018/8509583

**Published:** 2018-04-01

**Authors:** Camelia Frantz, Sonia Pezet, Jerome Avouac, Yannick Allanore

**Affiliations:** Inserm U1016, UMR8104, Rheumatology A Department, Cochin Hospital, Paris Descartes University, Paris, France

## Abstract

**Objective:**

To evaluate the performance of serum and urinary sCD163 concentrations as possible biomarker in systemic sclerosis (SSc).

**Methods:**

Urine and serum samples were obtained from SSc patients and age- and sex-matched controls. Serum and urinary sCD163 concentrations were measured by commercially available ELISA kit. SSc patients were assessed following international guidelines. Cross-sectional analyses were performed.

**Results:**

Two hundred and three SSc patients were included. The control group consisted of 47 age- and sex-matched patients having noninflammatory diseases, mainly osteoporosis. Serum sCD163 levels were significantly higher in SSc patients compared with controls (mean ± SD: 529 ± 251 versus 385 ± 153 ng/mL; *p* < 0.001). Urinary sCD163 concentrations were higher in SSc patients than controls, but this did not reach significance (236 ± 498 versus 176 ± 173 ng/mg uCr; *p* = 0.580). The sCD163 concentrations were not associated with clinical, laboratory, and instrumental characteristics of SSc patients.

**Conclusion:**

To our knowledge, this is the first evaluation of both serum and urinary sCD163 levels in SSc. Our results show a significant difference for sera values that should be prioritized for further studies as compared to urinary measurements. Our results further support that the M2 macrophages/CD163 signaling system may play a role in the pathogenesis of SSc, although we could not identify a subset of SSc patients with higher concentrations.

## 1. Introduction

Systemic sclerosis (SSc) is an orphan and incurable connective tissue disorder characterized by excessive collagen deposition in the dermis and internal organs, microvascular injury, and specific autoantibodies [1]. It is the most severe connective tissue disease, associated with high mortality risk [2]. Although the pathogenesis of the disease remains largely unknown, increasing evidence indicates that monocytes/macrophages play a key role in the development of both autoimmune and fibrotic diseases, such as SSc [3–5].

Macrophages are functionally distinguished as a classically M1-activated type that secretes high levels of proinflammatory cytokines and an alternatively activated M2 type, stimulated by Th2 cytokines, that exhibits an anti-inflammatory function and contributes to wound healing [6]. Although both M1 and M2 types seem to be involved in the pathogenesis of SSc, a strong M2 signature was observed in the skin, blood, and lungs of SSc patients [3].

CD163 is a type I transmembrane protein belonging to group B of the cysteine-rich scavenger receptor family that acts as a scavenger receptor for the haemoglobin-haptoglobin complex [7]. The expression of CD163 is constitutive and/or induced by some stimuli on circulating monocytes and most tissue macrophages, and it is a well-accepted marker for activated M2 macrophages [8]. A soluble form of human CD163 (sCD163) is released from the cell surface by proteolysis after oxidative stress or inflammatory stimuli.

Increased expression of CD163 on peripheral blood mononuclear cells and skin macrophages from patients with SSc as compared with healthy controls has been previously reported [9, 10]. Furthermore, several investigators have demonstrated increased sCD163 serum levels in patients with SSc compared with normal controls [11–13]. However, these studies had small sample size, due to the difficulties in recruiting large samples for such a rare condition. Moreover, to our knowledge, there has been no study examining the significance of urinary sCD163 levels in SSc although urinary concentrations are promising markers in other connective tissue diseases [14, 15].

We herein aimed to evaluate serum and urinary sCD163 levels in a large population of SSc patients and to assess any possible association with clinical, laboratory, and instrumental characteristics.

## 2. Materials and Methods

### 2.1. Patient and Sample Collection

Two hundred and three consecutive patients, fulfilling the 2013 American College of Rheumatology/European League Against Rheumatism classification criteria for SSc [16], were recruited from the Rheumatology Department of Cochin Hospital, Paris Descartes University. The control group consisted of 47 age- and sex-matched patients with noninflammatory diseases, being osteoporosis for the very large majority. Clinical and laboratory data were collected longitudinally from medical records since the time of serum and urinary samples were taken. Patients were classified as having diffuse cutaneous SSc (DcSSc) or limited cutaneous SSc (LcSSc) based on subset classification criteria [17]. Disease activity was measured through the European Scleroderma Study Group activity index (EScSG-AI) [18]. The following clinical data were collected for all patients: age, sex, disease duration (date of the first non-Raynaud symptom), skin involvement according to the modified Rodnan skin score (mRSS) [19], presence of interstitial lung disease (ILD) on high-resolution computed tomography (HRCT), pulmonary hypertension confirmed by right heart catheterization, digital ulcers, and treatment received. Pulmonary function was assessed based on measurements of forced vital capacity (FVC) and diffusing capacity of the lungs (DLCO) and expressed as percentage of values predicted. Laboratory data included Westergren erythrocyte sedimentation rate (ESR), C-reactive protein level (CRP), serum and urinary creatinine levels, and tests for anticentromere antibodies, antitopoisomerase I antibodies, and antipolymerase III antibodies.

Plasma and urine samples were stored at −80°C until use.

All patients and controls signed a consent form approved by the local institutional review boards (Comité de Protection des Personnes, Paris Ile de France 3).

### 2.2. Enzyme-Linked Immunosorbent Assay

Serum and urinary (u-) sCD163 concentrations were measured using enzyme-linked immunosorbent assay (ELISA) kits according to the manufacturer's instructions (DY1607 Duo Set; R&D Systems, Minneapolis, MN, USA). The detection range was 156–10,000 pg/mL. Serum samples were diluted 100-fold and urine samples were not diluted.

### 2.3. Statistical Analysis

Data were analyzed using Graphpad Prism software. Mann–Whitney *U* test was used for between-group comparisons. Spearman's rank correlation coefficient was used to examine the relationship between two continuous variables. All results were expressed as mean ± standard deviation (SD) unless stated otherwise. We considered *p* values less than 0.05 to be statistically significant.

## 3. Results

### 3.1. Patient Characteristics

A total of 203 SSc patients were included, of whom 163 (80%) were female. Mean ± SD age was 59 ± 13 years, and mean ± SD disease duration was 12 ± 9 years (since the first non-Raynaud's symptom). Eighty-one (41%) patients had diffuse cutaneous SSc and mean ± SD mRSS was 6.6 ± 7.7. Mean EScSG-AI score was 1.4 ± 1.7. ILD was observed in 33% of the patients, 7% had pulmonary arterial hypertension proven by right heart catheterization, 44% had history of digital ulcers, 41% were taking immunosuppressive therapy, and 38% were taking prednisone (mean ± SD 2.6 ± 4.3 mg/day). In the whole population, mean ± SD FVC and DLCO were 95 ± 37% and 64 ± 26%, respectively. Restrictive ventilatory defect (defined as FVC < 75% of what is predicted) and impaired diffusion capacity (defined as DLCO <75% of what is predicted) were found in 45% and 87% of patients with ILD, respectively. Mean ± SD pulmonary arterial systolic pressure (PASP) evaluated by Doppler echocardiography was 32 ± 9 mmHg. Antinuclear antibodies were detected in 184 SSc patients (91%); among whom, 68 (35%) were positive for anticentromere (ACA), 61 (31%) for anti-SCL70 autoantibodies, and 12 (6%) for antipolymerase III autoantibodies. Overall, laboratory tests showed normal to mild increased ESR and CRP values (mean ± SD: 18.7 ± 12.6 mm in the 1st hour and 5.5 ± 8.5 mg/L, resp.). Renal function was globally preserved (mean ± SD serum creatinine: 72 ± 33 mmol/L).

There was no significant difference in age and sex distribution between SSc patients and controls.

Table 1 shows the main demographic, clinical, and laboratory features of the study population.

### 3.2. Serum sCD163 Levels in SSc

**Figure 1:**
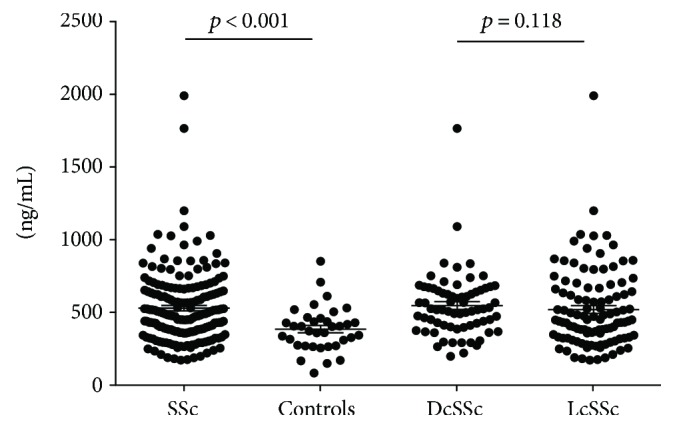
Serum concentrations of sCD163. Serum sCD163 levels were measured by ELISA in overall group of patients with systemic sclerosis (SSc), patients with diffuse SSc (DcSSc), those with limited SSc (LcSSc) and controls. Symbols show individual patients; bars show the mean ± SEM. *p* values were determined using Mann–Whitney *U* test.

Serum sCD163 levels were significantly increased in total SSc patients compared with controls (mean ± SD: 529 ± 251 versus 385 ± 153 ng/mL; *p* < 0.001) (Figure 1). The difference remained significant after removal of the two outliers in the SSc group (*p* = 0.0004). There were no significant differences in sCD163 levels between patients with DcSSc and those with LcSSc (mean ± SD: 547 ± 222 versus 519 ± 271 ng/mL; *p* = 0.484). No significant correlations between sCD163 levels and any clinical or laboratory variables could be found, including duration of the disease, mRSS, EScSG-AI score, FVC, DLCO, CRP, or ESR. Furthermore, when patients were subdivided according to disease duration, presence of lung fibrosis, pulmonary hypertension, digital ulcers, or specific autoantibodies, no significant difference was observed in sCD163 concentration. Also, serum sCD163 levels were not significantly influenced by ongoing immunosuppressive or prednisone therapy.

### 3.3. Urinary sCD163 Levels in SSc

**Figure 2:**
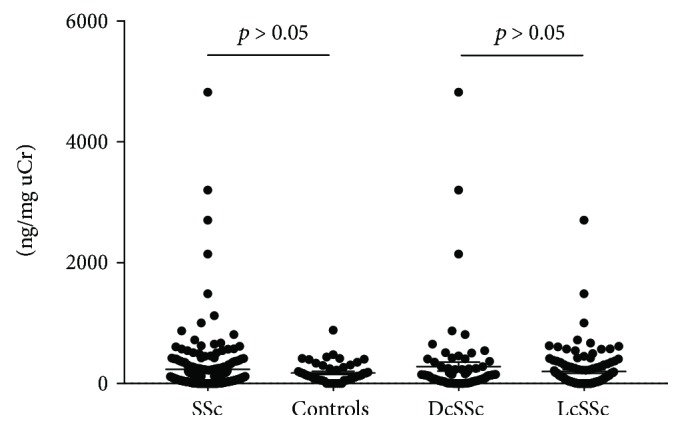
Urinary concentrations of sCD163, corrected by urinary creatinine (uCr). Urinary sCD163 levels were measured by ELISA and corrected by urinary creatinine (uCr) in the overall group of patients with systemic sclerosis (SSc), patients with diffuse SSc (DcSSc), those with limited SSc (LcSSc), and controls. Symbols show individual patients; bars show mean ± SEM. *P* values were determined using Mann–Whitney *U* test.

Urinary sCD163 concentrations in SSc patients were also higher than those in controls, but this did not reach significance (236 ± 498 versus 176 ± 173 ng/mg uCr; *p* = 0.580) (Figure 2). As well as for serum sCD163 levels, no subpopulation could be identified as having higher concentrations.

## 4. Discussion

Our findings indicate a significant difference in the serum concentration of sCD163 of SSc patients and controls suggesting that sCD163 might be a possible biomarker in SSc. This result also further supports that M2 macrophage activation and the CD163 signaling system may play a role in the pathogenesis of the disease. However, urine measurement does not seem to add to patient discrimination.

The concentrations observed in our series of patients and controls are in agreement with those found in the study of Shimizu et al. who used the same ELISA kit [11].

In our study, the sCD163 concentrations were not associated with clinical, laboratory, and instrumental characteristics of the disease. Notably, there were no significant differences in sCD163 concentrations between DcSSc and LcSSc patients. Our results are in agreement with those presented in previous published studies that could not show significant difference between the two main cutaneous subset [11–13]. Furthermore, increased expression of CD163 on peripheral blood mononuclear cells and skin macrophages from patients with SSc as compared with healthy controls have been reported but without significant associations between high expression and any phenotypic characteristic [9, 10]. One may suggest that early disease might be associated with higher macrophage activation but in our hands, we could not identify any difference with regards to the time of disease duration determined according to the first non-Raynaud's symptom. Even earlier stages should be targeted and VEDOSS patients should be investigated in the future [20].

Regarding clinical association, Hassan et al. and Nakayama et al. reported some associations between high serum concentrations of sCD163 and a vascular component of the disease that is elevated pulmonary artery pressure. In the herein study, values higher than mean + 2SD (Hassan et al.) or mean + 3SD (Nakayama et al.) of the control serum samples were used as cutoff points. Thus, Nakayama et al. reported higher prevalence of elevated PASP (>30 mmHg) in patients with elevated serum sCD163 levels than in those without (71.4 versus 28.6%, *p* < 0.05) [12]. Similarly, Hassan et al. reported higher PASP values in SSc patients with elevated serum sCD163 concentrations (53.22 ± 23.98 versus 37 ± 13.67 mmHg) [13]. It should be pointed out that these studies have limitations. First, the number of patients in the 2 studies was small (43 and 24, resp.), with only 8 and 9 patients, respectively, having elevated sCD163 levels. Secondly, the meaning of PASP with a cutoff of 30 mmHg could be challenged and it is well established that only right heart catheterization can measure adequately pulmonary pressure. In our study that included 203 patients (with 71/179 (39.6%) having PASP > 30, 22/179 (12.3%) having PASP > 40, and 14/192 (7.3%) with proven pulmonary hypertension), we could not find any relationship between sCD163 concentrations and lung vascular disease. Following the vascular phenotype [21], we could neither find any relationship between sCD163 concentrations and digital ulcers.

The use of urinary sCD163 as a potential biomarker has been investigated in systemic lupus erythematosus (SLE), especially in lupus nephritis [14] another connective tissue disease with a putative role of M2 macrophage. To our knowledge, our study is the first one evaluating urinary sCD163 levels in SSc. Our results show higher urinary sCD163 concentrations in SSc patients as compared to controls but the difference did not reach significance, suggesting that evaluation of serum sCD163 levels should be prioritized for further studies as compared to urinary concentrations. This also suggests that urinary sCD163 evaluation may be useful only in cases of kidney disease, such as lupus nephritis, explaining the difference between SLE and SSc.

## 5. Conclusion

To our knowledge, this is the first evaluation of both serum and urinary sCD163 levels in SSc. Our results show a significant difference for sera values that should be prioritized for further studies as compared to urinary concentrations conversely to what has been described in lupus. Moreover, larger and longitudinal studies will be required to determine the performance of sCD163 as a potential biomarker in SSc. Our results further support that alternatively, M2 macrophage activation and CD163 signaling system may play a role in the pathogenesis of the disease. However, further studies are required to address the exact role of CD163 in the pathogenesis of SSc and to determine whether it could help in the risk stratification of the patients in this heterogeneous disease.

## Figures and Tables

**Table 1:** Clinical characteristics of SSc patients and controls.

	SSc (*n* = 203)	Controls (*n* = 47)
Age (years)	58.9 ± 13.4	61.7 ± 13.9
Female, *n* (%)	163 (80.2)	43 (91.4)
Disease duration (years)	12 ± 8.9	—
Diffuse cutaneous subset, *n* (%)	81/199 (40.7)	—
Modified Rodnan skin score	6.6 ± 7.7	—
Modified Rodnan skin score > 14, *n* (%)	24/164 (17.7)	—
ILD by HRCT, *n* (%)	66/197 (33.5)	—
Pulmonary hypertension, *n* (%)	14/192 (7.3)	—
History of digital ulcers (past or present), *n* (%)	82/186 (44.1)	—
PASP (mmHg)	31.7 ± 9.2	—
PASP >30 mmHg, *n* (%)	71/179 (39.6)	—
PASP > 40 mmHg, *n* (%)	22/179 (12.3)	—
FVC (%pred.)	95 ± 37	—
FVC < 75%pred., *n* (%)	33/152 (21.7)	—
DLCO (%pred.)	64 ± 26	—
DLCO < 75%pred., *n* (%)	104/153 (68)	—
ACA positive, *n* (%)	68/194 (35)	—
Anti-Scl70 positive, *n* (%)	61/194 (31.4)	—
Anti-RNA polymerase III positive, *n* (%)	12/194 (6.2)	—
ESR (mm/h)	18.7 ± 12.6	—
CRP (mg/L)	5.5 ± 8.5	—
CRP > 10 mg/L, *n* (%)	22/145 (15.2)	—
Serum creatinine (*μ*mol/L)	72 ± 33	—
Serum creatinine > 120 *μ*mol/L, *n* (%)	5/132 (3.8)	—
Ongoing prednisone therapy, *n* (%)	66/172 (38%)	—
Ongoing immunosuppressive therapy, *n* (%)	67/164 (40.8)	—
EScSG-AI	1.4 ± 1.7	—
EScSG-AI > 3, *n* (%)	23/157 (14.6)	—
HAQ	0.7 ± 0.6	—

Data are expressed as mean ± SD unless stated otherwise. SD = standard deviation; ACA = anticentromere antibodies; CRP = C-reactive protein; DLCO = diffusing capacity of the lung; EScSG-AI = European Scleroderma Study Group activity index; ESR = erythrocyte sedimentation rate; FVC = forced vital capacity; ILD = interstitial lung disease; HAQ = Health Assessment Questionnaire; HRCT = high-resolution computed tomography; PASP = pulmonary arterial systolic pressure.

## Data Availability

All data from this study are available from the corresponding author on reasonable request.
